# Experimental Measurement of Emissivity of Polished Steel Strips from a Continuous Annealing Line

**DOI:** 10.3390/ma17133084

**Published:** 2024-06-23

**Authors:** Šimon Staško, Gustáv Jablonský, Augustín Varga, Róbert Dzurňák, Jan Kizek

**Affiliations:** 1 Institute of Metallurgy, Faculty of Materials, Metallurgy and Recycling, Technical University of Kosice, Letna 1/9, 042 00 Kosice, Slovakia; gustav.jablonsky@tuke.sk (G.J.); augustin.varga@tuke.sk (A.V.); robert.dzurnak@tuke.sk (R.D.); 2 Department of Process Technique, Faculty of Manufacturing Technologies with a Seat in Presov, Technical University of Kosice, Bayerova 1, 080 01 Presov, Slovakia; jan.kizek@tuke.sk

**Keywords:** emissivity, temperature, steel strip, thermal image, experimental measurement, thermal imaging camera

## Abstract

The long-term use of steel strip in various industries makes it an important semi-finished product, which makes it necessary to improve its chemical composition and mechanical properties, reduce its thickness and weight, expand the range of new types of steel strip and increase its production. This entails a large number of technological operations dependent on precise temperature measurement and control. In some industrial plants, the steel strip is in continuous motion, which makes the use of contact measuring devices impossible. When using non-contact measuring devices such as pyrometers or thermal imaging cameras, the emissivity of the materials being measured is a problematic parameter, as setting an incorrect emissivity value to the measuring device results in inaccurate temperature readings. The essence of this research was to establish a measurement method and to perform experimental measurements of the emissivity of a polished steel strip used in a continuous annealing line, the subsequent processing of the data from these measurements and their evaluation. The emissivity measurements were carried out for 5 types of steel strip of different parameters, while the measurement itself was carried out in the long wavelength range of 7.5–14 µm and at strip temperatures of 100–300 °C. Depending on the type of steel strip, the mean emissivity values ranged from 0.0835– to 0.1143. The emissivity of the steel strip increased with increasing strip temperature, and it was not a linear dependence. The emissivity values determined in this research could be applied to measuring equipment in actual production, which could improve the accuracy of temperature measurement in the heat treatment of polished steel strip. Thermal camera measurements in the long wavelength range, taking thermal images and their processing and determining the emissivity value of polished steel strips are the parts of this research that make it different from other already published research.

## 1. Introduction

Steel strips or sheets have long been an important input semi-finished product for the production of products in various industries. Depending on the industry, these strips have different chemical compositions. Every year, there is a demand to increase the production of these strips, to extend the range with new types of strips, to improve their properties and to reduce their thickness and weight [1]. In order to achieve these requirements, it is necessary to improve and innovate areas of technological and production processes. A large part of these processes takes place in industrial furnaces, which reach high outputs; thus, their energy consumption is significant [2].

Continuous annealing lines are among the important industrial furnaces designed for the heat treatment of cold-rolled steel strips. These annealing lines consist of several chambers in which the steel strip undergoes a significant temperature change in a short time. The first step is the heating of the strip in the heating chamber, in which this heating takes place mainly through heat transfer by radiation and partly by convection. In the next step, the steel strip remains at the annealing temperature in the annealing chamber, followed by its cooling in the cooling chambers [3,4]. In each chamber of the continuous annealing line, a gaseous protective atmosphere flows to protect the steel strip from oxidation [5,6]. The velocity of the steel strip movement in all the furnace chambers is synchronized with the heating and cooling rates of the strip. To ensure continuous operation, 2 unwinders are used in the inlet section and 2 winders are used in the outlet section. Once the desired length of strip has been wound, the strip is separated using the splitting shears. After annealing in this line, the deformed grains of the steel strip are replaced with new ones and thus its properties including ductility are improved [7].

To achieve the desired mechanical properties of the steel strip and to achieve higher energy and economic efficiency in the annealing process, precise control and the measurement of the temperature in the furnace and on the steel strip throughout the annealing process is necessary [8,9]. In most industries, contact measuring devices such as thermocouples are used for accurate temperature measurement. Since in a continuous annealing line, the steel strip is in continuous motion, it is problematic to measure its temperature by using contact measuring devices. In this case, it is practical to use pyrometers or thermal imaging cameras [10].

Infrared thermography is a branch of science dealing with the acquisition of thermal data from non-contact measuring devices and their subsequent processing. The basis of this thermography is infrared radiation, also referred to as thermal radiation, which is a part of electromagnetic radiation in the wavelength range from 0.76 µm to 1000 µm. The wavelengths of infrared radiation are longer than the wavelengths of visible light. The field of infrared radiation also includes industrial heat sources such as iron or steel production [11,12,13].

Infrared radiation is divided into several ranges according to the size of the wavelengths, and their exact values vary depending on the reference. Table 1 gives an overview of the different wavelength ranges according to multiple references [11,13].

There are other divisions; for example, according to the International Commission on Illumination (CIE) and the German Institute for Standardisation (DIN), infrared radiation is divided into 3 ranges [13,14]:

- IR-A—range from 0.78 µm to 1.4 µm
- IR-B—range from 1.4 µm to 3 μm
- IR-C—range from 3 µm to 1000 μm

There is a dependence between the radiated power and the temperature of the object, with a higher temperature resulting in a higher radiated power. Pyrometers and thermal imaging cameras are used for the non-destructive and non-contact measurement of the temperature or temperature field on the surface of an object emitting infrared radiation [11]. However, in the case of these non-contact measuring devices, for proper temperature measurement, it is necessary to know several parameters affecting the measurement, among which is the exact value of emissivity, which is an important parameter of heat transfer via the radiation of real objects [17].

Emissivity is a dimensionless parameter determining the ability of an object to emit thermal radiation. It is defined as the ratio between the radiated power of a real object and the radiated power of an absolute blackbody at the same temperature. The emissivity value ranges from 0 to 1 or rather 0 to 100% [18,19]. Based on Kirchhoff’s law, the emissivity of a material is equal to the absorptivity of the material, which is shown by Equation (1) [20]:

ε = α(1)

where ε represents emissivity and α represents absorptivity, which represents the ratio of radiation absorbed by the surface of the object.

For the absorptivity of non-transparent materials, Equation (2) is as follows:

α = 1 − ρ(2)

where ρ represents reflectivity, which represents the ratio of radiation reflected from the surface of the object [20].

The blackbody is an ideal emitter and represents 100% of the radiated power or intensity of thermal radiation [21]. The emissivity of the blackbody is equal to 1, which represents the maximum achievable value. In the case of a blackbody, it is assumed that at a steady temperature, all the absorbed radiation is emitted [11].

Emissivity has a significant impact on non-contact measurements by pyrometers or thermal imaging cameras. If the emissivity value set into the pyrometer or thermal imaging camera is different from that of the real measured object, then such a measurement would be accompanied by significant errors, which would distort, for example, the temperature measurement [22,23]. The correct emissivity value is also important for thermal engineering calculations and for creating mathematical models and process simulations. The current literature, scientific publications and other available resources provide emissivity values for various materials, including steel, with different types of steel having different emissivity values. In some cases, the emissivity value is different even for the same type of steel, or the emissivity value varies from steel strip to steel strip. As a result, there is a problem in selecting a suitable emissivity value and consequently, deviations arise in thermographic measurements and also in thermal engineering calculations and simulations. The emissivity of steel is affected by temperature, measurement wavelength range, chemical composition, surface roughness, oxidation, steel surface contamination, measurement angle and surface shape [24,25]. Another problem is that a large number of sources reporting the emissivity value for steel do not indicate under which conditions this value is valid, or the wavelength range and even the temperature are not specified. Steels with high chromium content mostly have a lower emissivity value than other types of steel; this is due to the protective chromium oxide layer [26].

The determination of emissivity using numerical simulations is very problematic and experimental measurement appears to be the most appropriate method for its determination [27]. The emissivity value of steel increases with increasing temperature, while the dependence in this case is not linear; this is evident from the results of experimental measurements reported in the scientific publications of the authors Roger et al., 1979 [28]; Wen, 2010 [26]; Wen, 2011 [10]; Woods et al., 2014 [29]; Zhang et al., 2015 [23]; Zareba et al., 2016 [30]; Deus et al., 2020 [31]; and Zhao et al., 2023 [32]. The emissivity of steel decreases with increasing wavelengths, and even in this case, it is not a linear dependence, as shown by the data in the scientific publications of the authors Wen, 2010 [26]; Wen, 2011 [10]; Xing et al., 2015 [33]; Fukuyama et al., 2019 [34]; Suleiman et al., 2021 [8]; Li et al., 2022 [35]; and Li et al., 2023 [36].

Table 2 shows the emissivity values of stainless steel at different wavelengths and temperatures.

The aim of this research is as follows:

- Determination of the method of measuring the emissivity of steel strips;
- Experimental measurement of the emissivity of steel strips, subsequent processing and evaluation of the measurement data.

## 2. Materials and Methods

The material for this experimental measurement was a polished steel strip used in continuous annealing. The surface of the polished steel strip has greater reflectivity than ordinary steel strips. Five types of this steel strip were selected for the experimental measurement, and the differences in the parameters of each type of strip were in thickness and quality. The parameters of the selected steel strip types are written in Table 3.

Equipment and software used during the experimental measurements:

- Electric laboratory muffle furnace VEB Elektro Bad Frankenhausen LM 212.11: AREKO s.r.o., Bratislava, Slovakia, temperature range of operation 0 to 1200 °C, dimensions 170 × 270 × 90 mm;
- Thermal imaging camera Testo 868: K-Test s.r.o., Košice, Slovakia, infrared resolution 160 × 120 pixels, spectral range 7.5 to 14 µm, measuring range 0 to +650 °C, accuracy ±2 °C, ±2% of measured value, emissivity 0.01 to 1;
- Thermocouple type K: Meratex s.r.o., Košice, Slovakia, 3 thermocouples for measuring the surface temperature of the polished steel strip, 1 thermocouple for measuring the temperature inside the furnace;
- Data logger Ahlborn ALMEMO 5690-1: AREKO s.r.o., Bratislava, Slovakia, for measured data acquisition;
- Thermo Hygrometer Beurer HM 16: NAY a.s., Bratislava, Slovakia, for measuring the ambient temperature and relative humidity around the laboratory furnace;
- Software ALMEMO control: version 5.20, for the read-out of the data logger;
- Software testo IRSoft: version 5.0, for processing thermal images.

The experimental measurement method was based on heating a polished steel strip to the required temperature of 100–300 °C and measuring the temperature of this strip using thermocouples and a thermal imaging camera. On both measuring devices, it was necessary to reach the same temperature value of the steel strip. The thermocouples were attached to the steel strip or touched it during the whole measurement and were therefore considered as an objective indication of the temperature of the steel strip. On the other hand, when the temperature was measured using a thermal imaging camera, it was possible to measure different temperature values of the steel strip. In order for the thermal imaging camera to be able to measure the same temperature of the steel strip as the thermocouples measured, it was necessary to set the correct emissivity value on the thermal imaging camera. The procedure of this measurement methodology was described in scientific publications by Rakrueangdet et al. 2016 [39] and Zhu et al. 2017 [40]. Since the adjustment of the emissivity to the thermal imaging camera in the real-time measurement was time-consuming, the last part of the measurement methodology was modified. During the experimental measurements, thermal images were taken of the steel strip and the emissivity determination was performed when these thermal images were processed using the software. In this way, the emissivity value for each type of steel strip was determined. This was followed by data processing and evaluation.

A steel strip temperature of 300 °C was chosen because the emissivity of steel increases with oxidation and the experimental measurement conditions did not have the protective atmosphere normally found in a continuous annealing line. At higher temperatures, the strip measurement would have been affected just by the oxidation of the steel strip surface, which was undesirable for this experimental measurement.

### 2.1. Procedure of Experimental Measurement

In the first step of each of the experimental measurements of this research, a data logger with a computer was run and the temperatures in the furnace and the temperatures on the surface of the steel strip were measured before the actual heating. Since no other measurements were made on this furnace outside of the experimental measurements, this first temperature measurement took approximately the same temperature value on all thermocouples, which represented the ambient temperature. The next step was to close the furnace door and start the electric laboratory furnace and set the heating to 130 °C. Thermocouples measured and recorded the change in temperature inside the furnace and on the surface of the strip the entire time, and these temperature changes showed that shortly after the heating was started, the temperature inside the furnace began to rise, as did the temperature on the steel strip. After the time required for heating had elapsed, the furnace automatically stopped heating the spirals, and these spirals, heated to a high temperature, continued to transfer heat to the furnace space, the steel strip, and the furnace walls. The temperature of the furnace space reached its maximum temperature of 130 °C and thereafter thermal exchange took place between the furnace space, the steel strip and the furnace walls until the temperatures were equalized.

**Figure 1:**
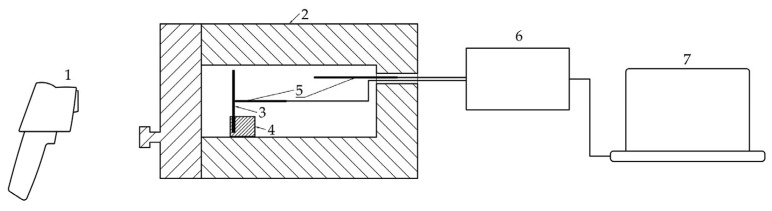
Schematic of the first part of the experimental measurement. (1) thermal imaging camera; (2) closed laboratory furnace; (3) polished steel strip; (4) bracket; (5) thermocouples; (6) data logger; (7) computer with temperature measurement software.

This first part of the measurement procedure is illustrated in Figure 1.

After the temperatures were equalized, or after the heat exchange between the furnace space, the steel strip and the furnace walls was completed, their temperature began to slowly decrease. When the temperature of the steel strip reached a value close to 100 °C, the furnace door was opened, and thermal images of the steel strip were taken with a thermal imaging camera; at the same time, the humidity and ambient temperature were recorded. The thermal imaging camera was placed on a tripod at a distance of 32 cm from the polished steel strip.

After the 100 °C thermal images were taken, two more heating periods followed, and thermal images of the steel strip were also taken at 200 °C and 300 °C. For each of these three temperatures, 3–5 thermal images were taken, which took 15–60 s to capture. At the end of the whole measurement, all the thermal images were transferred to the computer for processing. The measured temperatures from the data logger and the ambient temperatures were also stored.

**Figure 2:**
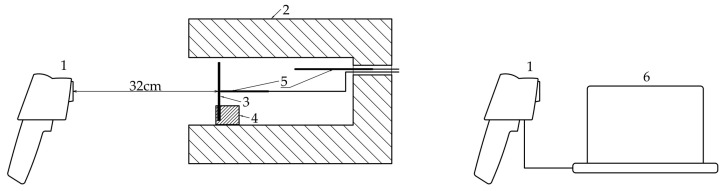
Schematic of the second part of the experimental measurement. (1) thermal imaging camera; (2) open laboratory furnace; (3) polished steel strip; (4) bracket; (5) thermocouples; (6) computer with software for processing of thermal images.

Figure 2 shows a schematic of the second part of the measurement procedure.

**Figure 3:**
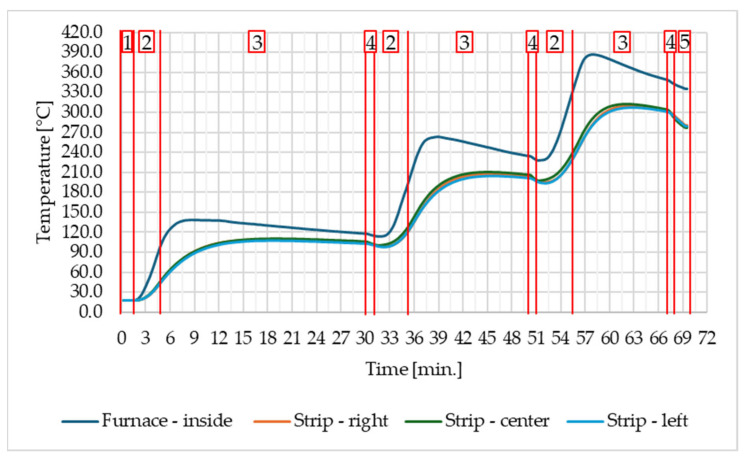
Temperatures in the furnace and on the steel strip throughout the measurement. 1. Switching on the data logger—recording the temperatures before heating; 2. closing the furnace door—starting the heating in the furnace; 3. switching off the furnace—transferring the heat to the temperature equilibration; 4. opening the furnace—taking thermal images; 5. leaving the door open and switching off the data logger.

Figure 3 shows the time dependence of the temperature measured by the thermocouples inside the furnace and on the surface of the steel strip throughout the measurement. This figure is also divided into sections according to the points indicated in the procedure of experimental measurement.

From Figure 3, it can be seen that the entire run of one of the measurements took 69 min, with the most time-consuming of the individual sections being the sections marked with the number 3, which shows the heat transfer inside the furnace. At the beginning of these sections, there is a significant temperature difference between the thermocouple measuring the temperature inside the furnace and the thermocouples measuring the temperature on the surface of the steel strip. The difference in these temperatures gradually decreases, but by the end of Section 3, these temperatures have ceased to equalize and all begin to decrease. On this basis, it was considered that there was a steady state in the furnace and the thermal images were taken.

**Figure 4:**
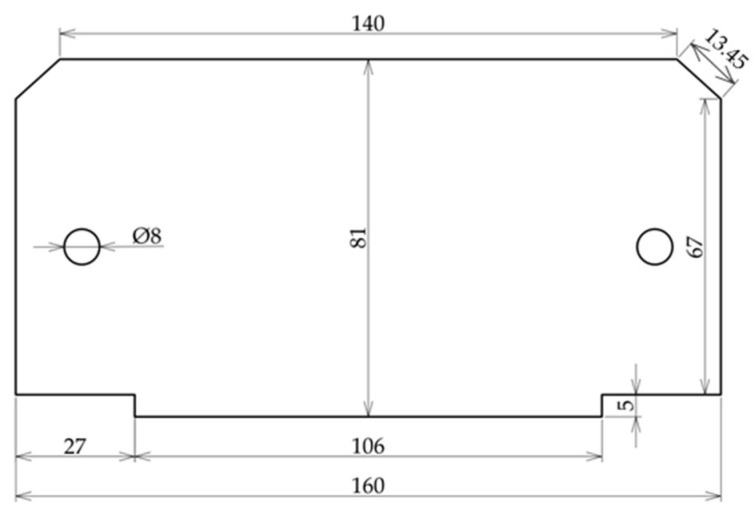
Dimensions of polished steel strip.

Figure 4 shows the dimensions of the steel strip, which were determined based on the dimensions of the laboratory furnace.

**Figure 5:**
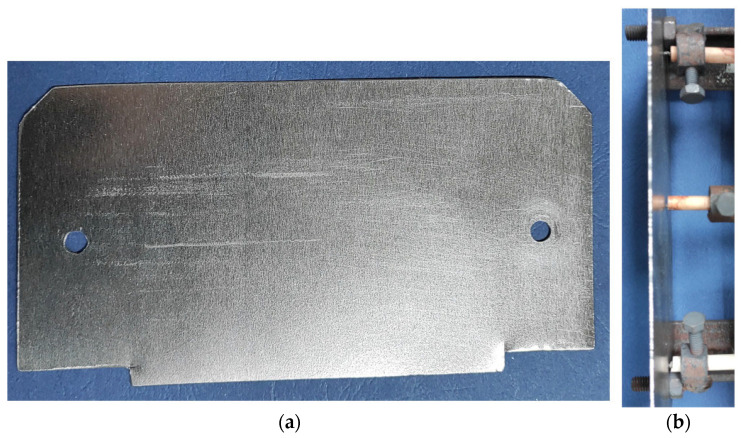
Polished steel strip. (**a**) Actual shape; (**b**) thermocouple location.

Figure 5 shows the actual shape of the polished steel strip and the location of the thermocouples measuring the temperature of this strip. The dimensions of the actual steel strip correspond to those shown in Figure 4 with an error of 1–2 mm due to the machining of the strip. The thermocouples were attached to the strip using screws placed in holes in the edges of the steel strip, and the thermocouple in the center of the strip was attached using a bracket.

Thermocouples attached to the steel strip had no protection and thus could be affected by the temperature of the hot air in the furnace. However, since a steady state condition was considered in the furnace just before the thermal images were taken, these thermocouple inaccuracies were neglected.

**Figure 6:**
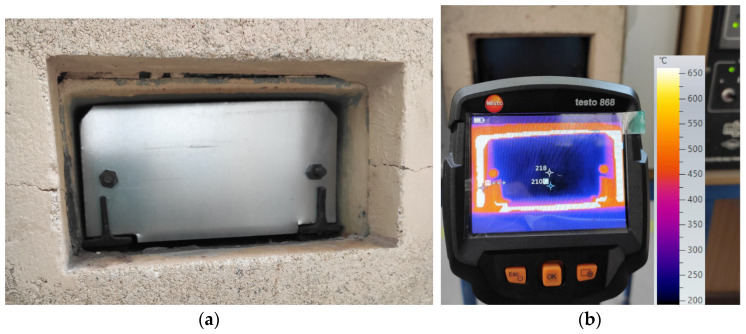
Taking thermal images of the steel strip. (**a**) Placement of the strip in the furnace area; (**b**) thermal imaging camera.

Figure 6 shows the placement of a polished steel strip in a laboratory furnace and the process of taking thermal images of this strip using a thermal imaging camera.

### 2.2. Data Processing

**Figure 7:**
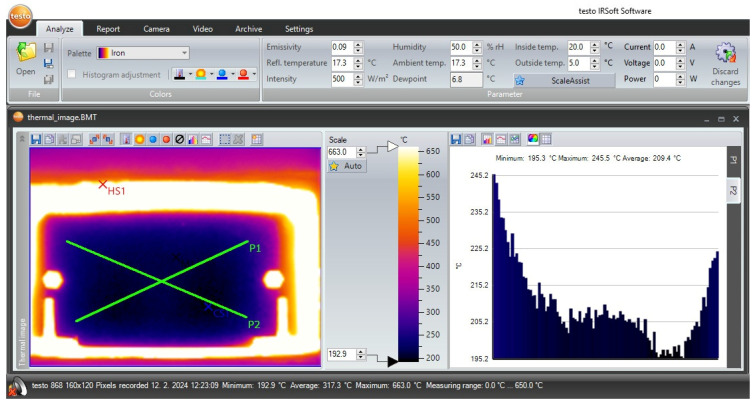
IRSoft software for processing thermal images from a thermal imaging camera.

One part of the data processing was to calculate the mean temperature of the steel strip as measured by the three thermocouples. The IRSoft software version 5.0 was used to process the thermal images, a screenshot of which is shown in Figure 7.

In this software, two diagonals were drawn across the steel strip surface showing the temperature progression of the steel strip along the length of the diagonals, and the temperature values from these diagonals were displayed on a graph in a side window of the IRSoft software. This graph showed the minimum, maximum and average temperature values of the steel strip. The average temperature of the steel strip in the IRSoft software was compared to the mean temperature measured by the thermocouples at the corresponding date and time when the thermal image was taken. The emissivity value was then determined via the emissivity window so that the average temperatures from the software and the thermocouples were the same or the difference was as small as possible. In most cases, it happened to be that two emissivity values were assigned to the average temperature of the steel strip. When adjusting the emissivity value in the software, the average temperature displayed in the software graph was in one case lower and in another case higher than the mean temperature measured by the thermocouples, and in both cases, the deviation was of a similar value.

During the imaging of a polished steel strip with a thermal imaging camera, it was possible to observe traces of infrared radiation on the surface of this strip from surrounding objects in the room with the door of the electric laboratory furnace open. These traces of infrared radiation were therefore also transferred to the thermal images, thus creating a temperature distortion on the steel strip. Corrections of such distorted temperatures were made just with the IRSoft software through the determination of the temperature value in the window reflected temperature. At the time of the experimental measurement, no activities were performed on the surrounding objects in the room and thus these objects had a temperature close to the ambient temperature value, so that in the processing of the thermal images, the ambient temperature was also considered as the reflected temperature at the same time.

In Figure 7, a weak temperature homogeneity of the steel strip can be observed. In the middle of the strip, the temperatures are lower and, on the other hand, at the edges of the steel strip, the temperatures reach the maximum values. It is assumed that this is the reflected temperature, while in the middle of the steel strip, the temperature from the room is reflected, and at the edges of the strip, the temperature from the front of the furnace walls is reflected. Since the exact value of the temperature reflected from the front of the furnace was not known, the diagonals were drawn mainly in the middle part of the strip where the reflected ambient temperature was considered in the processing of the thermal images.

During the continuous processing of the thermal images using IRSoft software, it was found that the measured humidity had no effect on the temperature change of the steel strip and therefore the humidity change was neglected when further thermal images were processed.

## 3. Results

The emissivity values from IRSoft software, the average temperatures of the steel strip measured by thermocouples and the ambient temperature values were processed by descriptive statistics. Specifically, the number of measurements, mean, standard deviation, minimum value, maximum value, and median were determined. These results from descriptive statistics are written in Table 4, Table 5, Table 6, Table 7 and Table 8.

**Figure 8:**
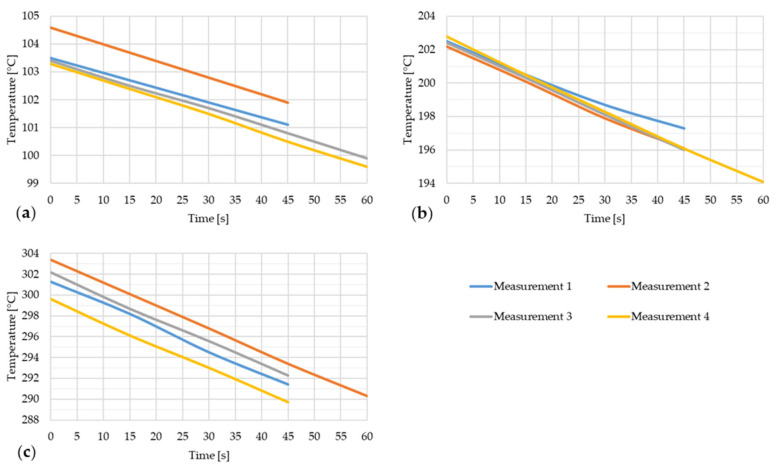
Temperature decrease of the steel strip during thermal image capture with the furnace door open for four measurements of the steel strip 1. (**a**) t ~ 100 °C; (**b**) t ~ 200 °C; (**c**) t ~ 300 °C.

The following figures show the time dependence of the temperature or the temperature decrease of the steel strip during the thermal image capture with an open furnace door. For each type of strip, the temperature dependences of four measurements are shown, which deal with the temperature decrease from the opening of the furnace door or from the taking of the first thermal image to the taking of the last thermal image of that measurement. The duration of these dependencies is in the range of 15–60 s, with the resulting time value depending on the specific measurement, as different numbers of thermal images were taken for each measurement. The first of these figures is Figure 8.

**Figure 9:**
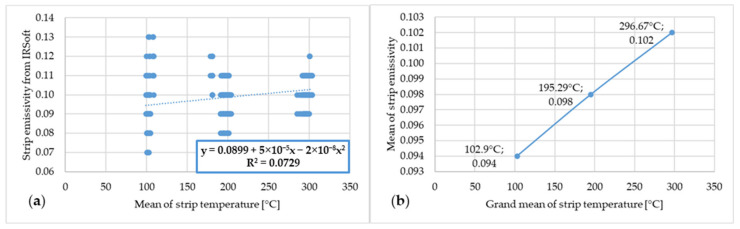
(**a**) Dependence of emissivity values from IRSoft software and mean temperature of steel strip 1; (**b**) dependence of mean emissivity value and grand mean temperature of steel strip 1.

The following figures marked with “a” show the dependence between the emissivity values from IRSoft software and the mean temperatures of the steel strip as measured by the thermocouples. The following figures marked with “b” show the dependence of the mean emissivity value and the grand average, or rather the mean of several means, of the steel strip temperature measured by the thermocouples. The first of these is Figure 9.

Figure 8 shows the temperature drop of steel strip 1 during thermal imaging.

**Table 4:** Descriptive statistics of measured data of steel strip 1. t1 ~ 100 °C; t2 ~ 200 °C; t3 ~ 300 °C.

Variable	*n*	Mean	Standard Deviation	Min	Max	Median
Steel strip emissivity t1	85	0.094	0.015	0.07	0.13	0.09
Steel strip temperature t1 (°C)	85	102.900	2.560	99.30	109.10	102.50
Ambient/reflected temperature t1 (°C)	85	17.480	1.589	13.80	18.80	18.00
Steel strip emissivity t2	85	0.098	0.009	0.08	0.12	0.10
Steel strip temperature t2 (°C)	85	195.297	6.372	178.70	203.95	196.90
Ambient/reflected temperature t2 (°C)	85	17.032	1.804	13.80	18.80	18.00
Steel strip emissivity t3	85	0.102	0.007	0.09	0.12	0.10
Steel strip temperature t3 (°C)	85	296.677	3.998	285.20	303.60	296.95
Ambient/reflected temperature t3 (°C)	85	17.103	1.756	13.80	18.80	18.00

Figure 9 shows the dependence of emissivity and temperature of steel strip 1.

In the graph, or rather in Figure 9a, a large range of measured emissivity values of 0.07–0.13 can be observed at 100 °C. This range decreases as the steel strip temperature increases, and at a strip temperature of 300 °C, the measured emissivity value is in the range of 0.09–0.12. The lower limit of the measured emissivity value increased with increasing temperature, and conversely, the higher limit of the measured emissivity decreased. Based on the literature review, it is assumed that this measurement instability at lower temperatures is due to the lower radiated power at which the thermal imaging camera was unable to measure with the necessary accuracy. As the temperature increased, the radiated power increased and thus the ability of the thermal imaging camera to measure a more accurate emissivity value increased. A similar trend in the measured emissivity values can be observed in the other graphs or figures marked with “a”.

**Figure 10:**
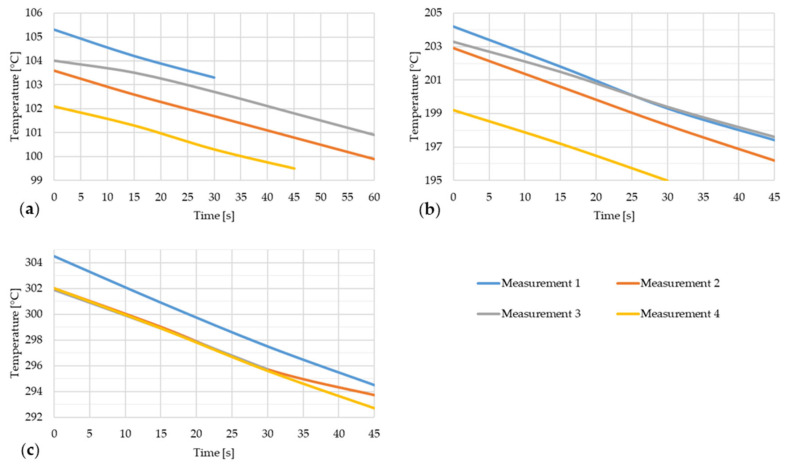
Temperature decrease of the steel strip during thermal image capture with the furnace door open for four measurements of the steel strip 2. (a) t ~ 100 °C; (b) t ~ 200 °C; (c) t ~ 300 °C.

Figure 10 shows the temperature drop of steel strip 2 during thermal imaging.

**Table 5:** Descriptive statistics of measured data of steel strip 2. t1 ~ 100 °C; t2 ~ 200 °C; t3 ~ 300 °C.

Variable	*n*	Mean	Standard Deviation	Min	Max	Median
Steel strip emissivity t1	75	0.086	0.013	0.06	0.11	0.09
Steel strip temperature t1 (°C)	75	102.230	2.679	97.15	107.00	102.10
Ambient/reflected temperature t1 (°C)	75	17.966	1.195	14.30	18.90	17.90
Steel strip emissivity t2	75	0.088	0.008	0.07	0.11	0.09
Steel strip temperature t2 (°C)	75	197.614	3.567	189.55	202.75	198.15
Ambient/reflected temperature t2 (°C)	75	18.009	1.172	14.40	18.90	18.60
Steel strip emissivity t3	75	0.092	0.007	0.08	0.11	0.09
Steel strip temperature t3 (°C)	75	298.262	2.244	294.05	302.65	297.95
Ambient/reflected temperature t3 (°C)	75	17.860	1.323	14.30	18.90	17.90

**Figure 11:**
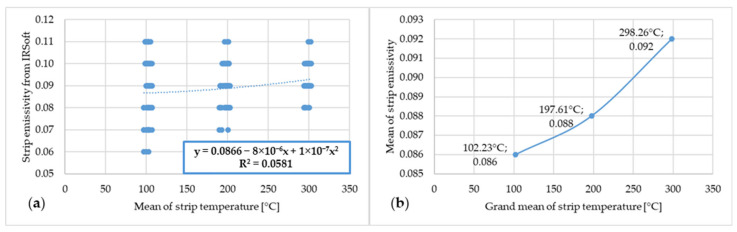
(**a**) Dependence of emissivity values from IRSoft software and mean temperature of steel strip 2; (**b**) dependence of mean emissivity value and grand mean temperature of steel strip 2.

Figure 11 shows the dependence of emissivity and temperature of steel strip 2.

**Figure 12:**
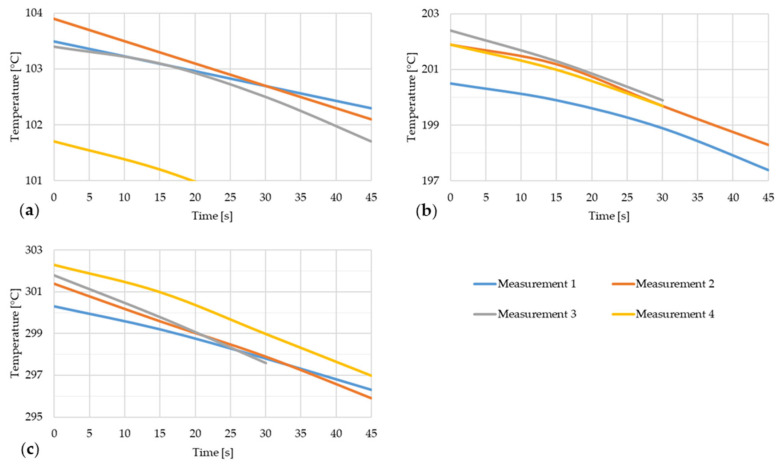
Temperature decrease of the steel strip during thermal image capture with the furnace door open for four measurements of the steel strip 3. (**a**) t ~ 100 °C; (**b**) t ~ 200 °C; (**c**) t ~ 300 °C.

Figure 12 shows the temperature drop of steel strip 3 during thermal imaging.

**Table 6:** Descriptive statistics of measured data of steel strip 3. t1 ~ 100 °C; t2 ~ 200 °C; t3 ~ 300 °C.

Variable	*n*	Mean	Standard Deviation	Min	Max	Median
Steel strip emissivity t1	80	0.100	0.013	0.08	0.13	0.10
Steel strip temperature t1 (°C)	80	103.353	2.536	98.10	106.85	103.30
Ambient/reflected temperature t1 (°C)	80	19.711	0.356	18.90	20.00	19.90
Steel strip emissivity t2	80	0.108	0.010	0.09	0.13	0.11
Steel strip temperature t2 (°C)	80	201.400	2.411	197.30	206.45	200.57
Ambient/reflected temperature t2 (°C)	80	19.742	0.331	18.90	20.00	19.90
Steel strip emissivity t3	80	0.114	0.008	0.09	0.13	0.11
Steel strip temperature t3 (°C)	80	300.365	5.194	287.95	306.15	301.50
Ambient/reflected temperature t3 (°C)	80	19.707	0.355	18.90	20.00	19.90

**Figure 13:**
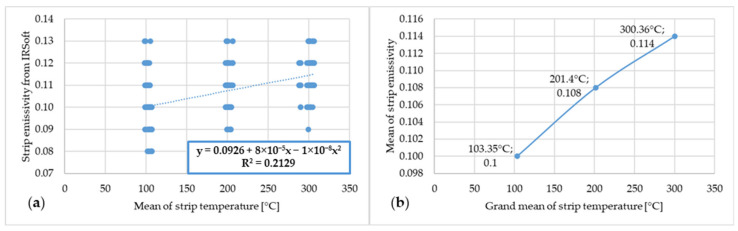
(**a**) Dependence of emissivity values from IRSoft software and mean temperature of steel strip 3; (**b**) dependence of mean emissivity value and grand mean temperature of steel strip 3.

Figure 13 shows the dependence of emissivity and temperature of steel strip 3.

**Figure 14:**
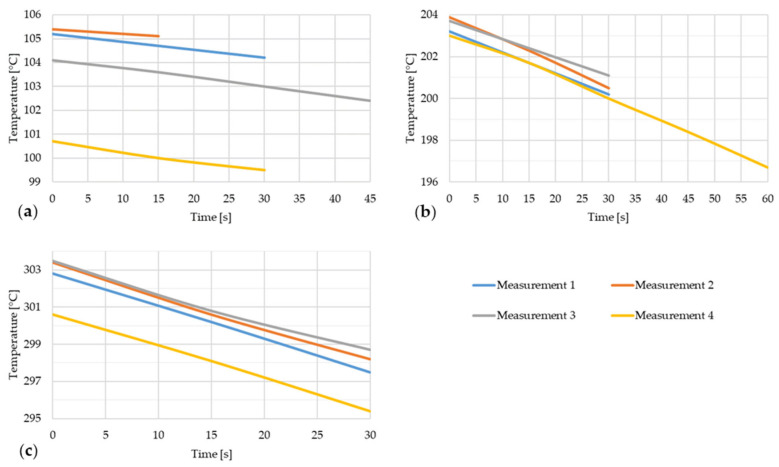
Temperature decrease of the steel strip during thermal image capture with the furnace door open for four measurements of the steel strip 4. (a) t ~ 100 °C; (b) t ~ 200 °C; (c) t ~ 300 °C.

Figure 14 shows the temperature drop of steel strip 4 during thermal imaging.

**Table 7:** Descriptive statistics of measured data of steel strip 4. t1 ~ 100 °C; t2 ~ 200 °C; t3 ~ 300 °C.

Variable	*n*	Mean	Standard Deviation	Min	Max	Median
Steel strip emissivity t1	65	0.083	0.011	0.06	0.11	0.08
Steel strip temperature t1 (°C)	65	103.934	2.122	99.10	107.65	104.20
Ambient/reflected temperature t1 (°C)	65	16.661	1.053	14.90	18.70	17.30
Steel strip emissivity t2	65	0.087	0.008	0.07	0.10	0.09
Steel strip temperature t2 (°C)	65	202.150	2.201	197.00	206.45	201.95
Ambient/reflected temperature t2 (°C)	65	16.967	1.037	14.90	18.70	17.30
Steel strip emissivity t3	65	0.090	0.007	0.08	0.11	0.09
Steel strip temperature t3 (°C)	65	299.816	2.484	294.90	303.40	299.70
Ambient/reflected temperature t3 (°C)	65	16.967	1.070	14.90	18.70	17.30

**Figure 15:**
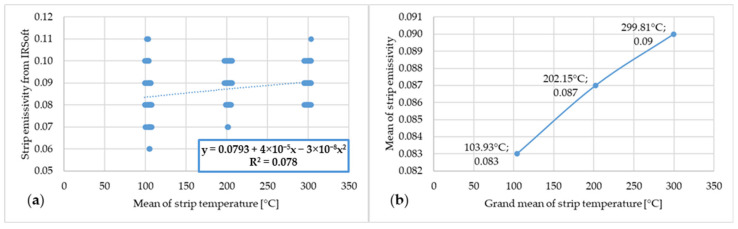
(**a**) Dependence of emissivity values from IRSoft software and mean temperature of steel strip 4; (**b**) dependence of mean emissivity value and grand mean temperature of steel strip 4.

Figure 15 shows the dependence of emissivity and temperature of steel strip 4.

**Figure 16:**
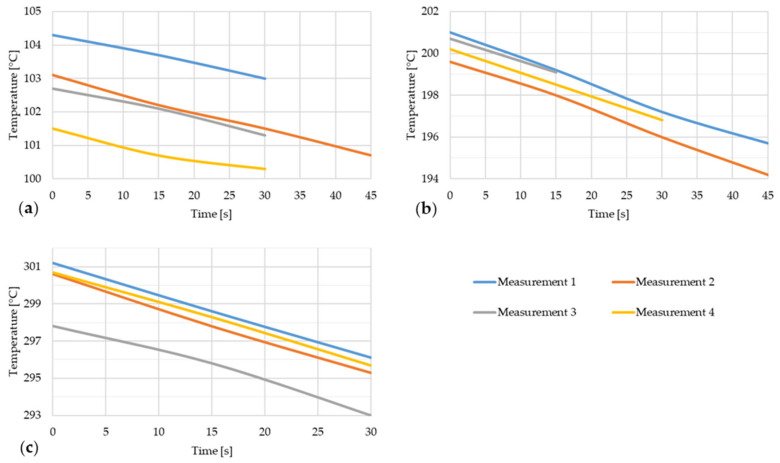
Temperature decrease of the steel strip during thermal image capture with the furnace door open for four measurements of the steel strip 5.

Figure 16 shows the temperature drop of steel strip 5 during thermal imaging.

**Table 8:** Descriptive statistics of measured data of steel strip 5. t1 ~ 100 °C; t2 ~ 200 °C; t3 ~ 300 °C.

Variable	*n*	Mean	Standard Deviation	Min	Max	Median
Steel strip emissivity t1	60	0.086	0.012	0.06	0.11	0.09
Steel strip temperature t1 (°C)	60	103.315	2.309	98.10	107.60	103.42
Ambient/reflected temperature t1 (°C)	60	18.850	0.508	18.20	19.50	19.10
Steel strip emissivity t2	60	0.089	0.009	0.07	0.11	0.09
Steel strip temperature t2 (°C)	60	199.380	2.557	194.45	204.00	199.70
Ambient/reflected temperature t2 (°C)	60	18.820	0.505	18.20	19.50	19.10
Steel strip emissivity t3	60	0.094	0.005	0.08	0.11	0.09
Steel strip temperature t3 (°C)	60	296.976	2.092	292.85	300.15	297.05
Ambient/reflected temperature t3 (°C)	60	18.826	0.487	18.20	19.50	19.10

**Figure 17:**
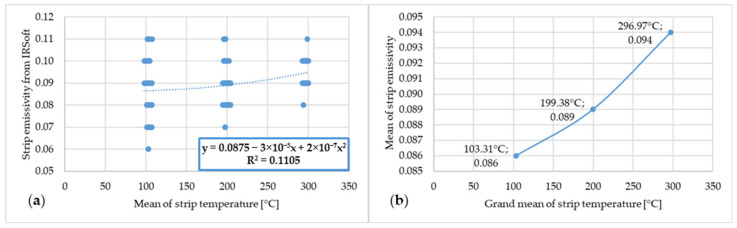
(**a**) Dependence of emissivity values from IRSoft software and mean temperature of steel strip 5; (**b**) dependence of mean emissivity value and grand mean temperature of steel strip 5.

Figure 17 shows the dependence of emissivity and temperature of steel strip 5.

## 4. Discussion

The measured emissivity values from this experimental measurement are constrained to thermal imaging camera wavelengths in the range 7.5–14 µm, which corresponds to the long wavelength range. The processing of the measured data using descriptive statistics yielded the following results:

- The mean temperature of the steel strip measured by the thermocouples is in the range 102.23–300.36 °C.
- The range of the mean ambient temperature is 16.66–19.74 °C.
- The mean emissivity value of the five steel strip types is in the range 0.083–0.114, with the lowest mean emissivity value of 0.083 with a standard deviation of 0.011 belonging to steel strip 4 and the highest mean emissivity value of 0.114 with a standard deviation of 0.008 from the measurements of steel strip 3. In terms of the general emissivity range of 0–1, or the range 0–100%, the emissivity results of the steel strip in this experimental measurement are near its lower limit, which represents a significantly low value.

In the figures showing the dependence of emissivity and temperature it is possible to see the increase in emissivity value with increasing temperature of the steel strip. This dependence between emissivity and steel strip temperature is not linear but polynomial. These results correspond to the results of the scientific publications presented in the introduction chapter of this article.

### 4.1. Emissivity Difference of the Same Grade of Steel

In Table 4, Table 5, Table 6, Table 7 and Table 8, different emissivity values can be observed between all types of steel strip. This is mainly due to the different steel grades of these strips, and the thickness of the strips also has some influence.

Steel strip 4 and steel strip 5 have the same grade value, so that in their case, the difference in emissivity is only due to the thickness of the strip. The difference in their emissivity at 100 °C is 0.003, at 200 °C it is 0.0016 and at 300 °C the difference in their emissivity is 0.0043. In all three cases, the higher emissivity value belongs to steel strip 5, which is 0.028 mm thicker than steel strip 4. From this comparison, it is clear that the strip with the greater thickness achieves a higher emissivity value than the thinner strip at the same grade and temperature. It is assumed that for the thicker strip to reach the same temperature as the thinner strip, it must accept a greater amount of heat. The same must also be true in reverse, so that when the furnace door was opened, the thicker strip cooled slower, and at the time when the thermal images were taken, it may have been at a slightly higher temperature than the thinner strip. However, in this case, there is no significant difference in emissivity.

### 4.2. Comparison of References and This Research

A common feature of the references and this research is the same basis of measurement methodology. Specifically, this research conducted the search for the same temperature of the object under investigation by using thermocouples and at the same time using a thermal imaging camera, whereby the same temperature can be achieved just by setting the appropriate emissivity value in the thermal imaging camera.

The difference is, for example, that in the case of the sources investigated, the emissivity measurements were in most cases made in the short wavelength range, whereas under the conditions of this research, the emissivity was measured in the long wavelength range. Another difference is that some sources measured the emissivity during the oxidation of the steel, but in this research, the oxidation of the steel strip was unacceptable, and the measurement was performed without oxidation. Under the conditions of this research, thermal images were taken during the measurement and processed as follows; this method was not presented in the sources reviewed. At the same time, during the search for sources, it was found that not many scientific sources deal with the issue of polished steel strips, and this is where the contribution of this research lies, as the results of the emissivity values of these strips are presented in this article.

## 5. Conclusions

Steel strips have long been an important input semi-finished product for several industrial areas; therefore, it is necessary to improve their chemical and mechanical properties, reduce their thickness and weight, expand the range of new types of steel strips and increase their production. In order to meet these requirements, the steel strip has to go through a large number of technological operations in which the accuracy of temperature measurement and control is crucial. In industrial plants where the steel strip is in constant motion, it is practically impossible to measure temperature using contact measuring devices. When using non-contact measuring devices such as pyrometers or thermal imaging cameras, the unknown emissivity value of the materials being measured is a problem, as setting the wrong emissivity value to the measuring device results in distorted temperature measurement data. Under the conditions of this research, the method of measurement was established and subsequently experimental measurements of the emissivity of a polished steel strip used in a continuous annealing line were carried out. This was followed by the processing of the data from these measurements and their evaluation. The experimental measurement of emissivity was carried out for five types of steel strips of different grades and thicknesses, the measurement itself being carried out in the temperature range 100–300 °C. Thermal images for emissivity determination were captured by a thermal imaging camera operating in the spectral range 7.5–14 µm, which represents the long wavelength range. After processing the measured data using descriptive statistics, the mean emissivity values ranged from 0.0835 to 0.1143 depending on the type of steel strip. The experimental results showed that the emissivity value of the steel strip increased as the temperature measured on the surface of this strip increased. The dependence between emissivity and strip temperature was not linear. In the case of actual production, the resulting emissivity values from this research could be applied to measuring equipment of the appropriate wavelength, which could improve the accuracy of temperature measurement of polished steel strips.

## Figures and Tables

**Table 1:** Wavelength ranges by multiple references.

Wavelength Range	Abbrevation	Value of Wavelengths by Reference (μm)			
		Usamentiaga et al., 2014 [11]	InfraTec [14]	RP Photonics Encyclopedia [15]	ISO 20473 [16]
Near-infrared	NIR	0.8–1.7	0.78–1.4	0.75–1,4	0.78–3
Short-wavelength infrared	SWIR	1–2.5	1.4–3	1,4–3	-
Mid-wavelength infrared	MWIR	2–5	3–8	3–8	3–50
Long-wavelength infrared	LWIR	8–14	8–15	8–15	-
Far-infrared	FIR	-	15–1000	15–1000	50–1000

**Table 2:** Emissivity of stainless steel.

Wavelength (µm)	Temperature (°C)	Emissivity	Reference
1	-	0.350	[37]
1	500	0.436	[23]
1	700	0.651	[23]
1.6	-	0.250	[37]
2	500	0.424	[23]
2	700	0.605	[23]
8	−60	0.131	[38]
10	−60	0.149	[38]
12	−60	0.125	[38]
14	−60	0.112	[38]
8–14	-	0.100	[37]

**Table 3:** Parameters of polished steel strip.

Variable	Steel Strip 1	Steel Strip 2	Steel Strip 3	Steel Strip 4	Steel Strip 5
Steel strip grade	581	589	582	549	549
Steel strip thickness (mm)	0.48	0.47	1.38	0.52	0.80

## Data Availability

The original contributions presented in the study are included in the article, further inquiries can be directed to the corresponding author.
